# Boosting work engagement through leader tolerance: the chain mediation role of perceived organizational support and organizational identification

**DOI:** 10.3389/fpsyg.2025.1489147

**Published:** 2025-03-12

**Authors:** Yuan Zhang, Jingqun Zhang, Kui Hao

**Affiliations:** ^1^Business School, Zhuhai College of Science and Technology, Zhuhai, China; ^2^Outpatient Department, Zaozhuang Municipal Hospital, Zaozhuang, China

**Keywords:** leader tolerance, work engagement, affective events theory, perceived organizational support, organizational identification

## Abstract

**Introduction:**

To enhance competitiveness, numerous organizations have introduced control and penalty systems to manage employee work errors. However, these systems have often backfired, negatively impacting employees’ emotions and behaviors. Recognizing the critical role of leadership in error management, this study examines how leaders’ tolerance of their followers’ mistakes influences employees’ work engagement, drawing on Affective Events Theory (AET).

**Methods:**

Analyzing data from 435 front-line public health service staff, this study investigates the relationship between leader tolerance and employees’ work engagement. First, the Harman one-factor test was employed to assess common method variance (CMV) in the research data. Second, the reliability and validity of the data were evaluated using the Cronbach’s α, KMO, AVE, CR, and CFA. Finally, the proposed mediating hypotheses were tested using Model 6 in the SPSS Process macro (version 4.1).

**Results:**

We found that leader tolerance significantly boosts employees’ work engagement. Furthermore, our results confirm the mediating roles of perceived organizational support (POS) and organizational identification in the relationship between leader tolerance and work engagement. This study also validates the hypothesized chain mediation model, demonstrating how POS and organizational identification together mediate the influence of leader tolerance on employees’ work engagement.

**Discussion:**

These results underscore the importance of leadership styles that accommodate employees’ errors and emphasize the crucial roles of organizational support and identification. The findings highlight the need for organizations to adopt more supportive leadership approaches rather than relying solely on control and penalty systems. The study concludes by stating the theoretical and practical implications, along with recommendations for future research on leader tolerance.

## 1 Introduction

Workplace errors, defined as unintentional deviations from goal achievement, can jeopardize organizational performance (Reason, 1995; Hofmann and Frese, 2011). Despite the inevitability of these errors (Chen et al., 2021), organizations have implemented strict error prevention measures to mitigate adverse outcomes (Frese and Keith, 2015), such as the widely adopted “zero-tolerance policy,” especially in the public health service industry. This policy enforces strict accountability for any mistakes, often leading to increased workloads, heightened psychological strain, and reduced job satisfaction, which can ultimately hinder organizational growth.

However, errors can also have positive effects, such as enhancing employees’ task competence and adaptability (Bell and Kozlowski, 2008). Several scholars advocate for error management strategies that promote open communication, thorough analysis, and corrective actions (Frese and Keith, 2015; Dimitrova and Van Hooft, 2021). Notably, leadership performs a vital role in this approach, as leaders’ attitudes and decisions significantly influence how organizations respond to errors (Van Dyck et al., 2013). Leader tolerance, defined as the willingness to excuse employees’ minor mistakes (Tang et al., 2015), has emerged as a crucial factor, with research indicating its profound impact on employees’ attitudes and behaviors.

Existing research, primarily grounded in Social Information Processing theory (SIP), suggests that leader tolerance fosters proactive employee behaviors by creating initiative climate and psychological safety (Zhou and Cheng, 2020). It motivates employees to reflect on their errors and strengthens their resilience (Zhang et al., 2024). Moreover, leader tolerance can enhance subordinates’ psychological ownership by improving leader-member exchange (Cao et al., 2024).

While Zhou and Cheng (2020) have shown that leader tolerance in nurturing employee proactivity, there is limited empirical evidence connecting this concept to employees’ work attitudes and behaviors. Also, work engagement, a critical indicator of work attitude, serves as a strong predictor of pro-organizational behavior (Bledow et al., 2011). Additionally, fostering work engagement can help organizations become more adaptable and flexible, enabling them to better navigate the uncertainty and complexity of external environments.

In this context, this research aims to contribute theoretically by proposing a conceptual model that links leader tolerance with employees’ work engagement. Building on Affective Events Theory (AET) and existing literature, the research examines how leader tolerance can enhance subordinates’ work engagement by sequentially influencing their perceived organizational support (POS) and organizational identification.

Unlike previous studies that have primarily focused on enterprise employees, we target a sample of Chinese hospital staff. In recent years, medical professionals have faced exceptionally high occupational stress and increased professional risks, along with the need to stay current with rapidly advancing medical technologies. In this high-pressure environment, strict error prevention policies can intensify heightened anxiety, leading to feelings of abandonment and mistrust among staff. This atmosphere may encourage individuals to conceal their mistakes or disengage from their work, ultimately harming both individual well-being and organizational development.

Understanding the influence of leader tolerance on employee engagement is crucial in this context. Our research aims to deepen our understanding of leader tolerance and provide practical insights specifically tailored for the effective error management in high-pressure industries. The results emphasize the importance of managing employees’ work errors effectively to foster work engagement.

## 2 Theory and hypotheses

### 2.1 Leader tolerance

Leader tolerance denotes the degree to which leaders are willing to excuse non-principled errors made by their subordinates, thereby fostering a learning environment (Cao et al., 2024; Zhang et al., 2021). Comparatively, leaders who exhibit high tolerance levels are more likely to forgive mistakes without imposing harsh penalties, while also promoting a culture of learning by offering constructive feedback and necessary resources (Zhou and Cheng, 2020). In contrast, leaders with low tolerance create a stressful work environment, characterized by strict supervision, frequent criticism, and punishment (Zhou and Cheng, 2020). These leaders often fail to provide the support and guidance needed when errors occur.

Taken together, the study of leader tolerance has often been overshadowed by the broader concept of inclusive leadership, which aims to foster belongingness and appreciation inside teams (Carmeli et al., 2010). Inclusive leadership is characterized by three key attributes: accessibility, availability and openness, which together foster a respectful and inclusive atmosphere for team members (Carmeli et al., 2010). While both leader tolerance and inclusive leadership share the common goal of promoting a positive organizational environment and performance, they differ in focus (Tang et al., 2015). Therefore, leader tolerance specifically addresses how leaders handle employees’ errors (Tang et al., 2015), whereas inclusive leadership emphasizes leveraging team diversity to achieve synergistic outcomes (Carmeli et al., 2010; Shore and Chung, 2022).

Besides, it also differs from other broader leadership construct. Compared with servant leadership which prioritize empowering subordinates holistically (Eva et al., 2019), leader tolerance specifically mitigates fear of blame after errors, crucial in high-stakes healthcare settings. And for learning-oriented leadership, both encourage error reporting, but leader tolerance emphasizes post-error support, for instance, non punitive actions, whereas learning-oriented leadership focuses on pre-error skill-building, for instance, training (Wallo et al., 2024), and transformational leaders inspire their followers through vision and charisma but may unintentionally stigmatize failures (Bass and Avolio, 1990). In contrast, leader tolerance explicitly normalizes errors as improvement opportunities.

Empirical research regarding the impact of leadership tolerance have yielded inconsistent outcomes. Most studies agree that leader tolerance positively influences employee behavior, with evidence showing it can enhance proactive customer service by fostering both initiative and psychological safety at group and individual levels (Zhou and Cheng, 2020). Leader tolerance has also been associated with improved error reflection and resilience among employees, as suggested by SIP theory (Zhang et al., 2024). Nevertheless, some studies reveal that leader tolerance may inadvertently lead to green silence behavior, where employees refrain from speaking up about environmental issues. This occurs through the parallel mediation of psychological ownership and moral disengagement, potentially hindering long-term organizational sustainability (Cao et al., 2024).

A significant limitation in current research is its heavy emphasis on the cognitive impacts of leader tolerance, often framed through SIP theory, while largely overlooking the affective experiences of employees. This exclusive concentration on cognitive factors has led to an inadequate comprehension of the complex interplay between leaders being tolerant and the behaviors of employees.

The perception of leader tolerance may vary across different culture. In collectivist culture, where the emphasis is on group harmony and interdependence (Oyserman and Lee, 2008), leader tolerance may be more readily accepted and valued by employees. Employees in such culture may regard leader tolerance as an indication of their leaders’ empathy and encouragement, which can enhance their sense of inclusion to the organization. In contrast, in individualist culture, leader tolerance may indeed be perceived as a form of loose management. Given that employees in such culture tend to focus more on personal values rather than collective values and interests (Oyserman and Lee, 2008), leader tolerance can trigger negative impacts such as a decline in work efficiency and an increase in counterproductive work behavior (CWB).

Against such background, SIP theory has paid limited attention to the context of how leader tolerance influence employees’ behavior, restricted the reflection of the actual situation. In comparison, AET theory is more sensitive to cultural differences in the perception and impact of leader tolerance. It recognizes that emotions and their expression vary across cultures, and take these differences into account when examining the relationship between leader tolerance and employee behavior. This helps to overcome the cultural bias that may be present in SIP theory.

To address these gaps, AET is introduced as a complementary framework that provides a more nuanced, dynamic and accurate perspective on the connection between leader tolerance and employees’ work engagement.

### 2.2 Affective events theory

AET offers a robust framework for understanding the immediate emotional responses to work-related events and how these emotions subsequently impact employees’ attitudes and behaviors (Weiss and Cropanzano, 1996). According to AET, work events are specific incidents within the workplace that have the potential to trigger emotional shifts among employees (Weiss and Cropanzano, 1996). The concept was further developed by identifying five types of affective work events: leaders’ behaviors, working conditions, group characteristics, stressful incidents, and organizational rewards and punishments (Brief and Weiss, 2002). This framework highlights the need for considering emotional responses when analyzing the impact of leader tolerance on employee engagement.

AET describes a causal chain where specific work events serve as immediate triggers for emotional responses, which then lead to behavioral changes (Weiss and Cropanzano, 1996; Ashkanasy and Dorris, 2017). Unlike other models that focus on broad environmental factors or individual traits, AET emphasizes the impact of particular work events. It suggests that employees’ behaviors are better predicted by their emotional reactions to these events than by stable workplace attributes (Weiss et al., 1999; Fisher, 2000). Furthermore, AET also distinguishes between affect-driven behaviors, which are spontaneous and emotion-based, and judgment-driven behaviors, which are more deliberate and based on rational evaluation (Weiss and Cropanzano, 1996).

Moreover, AET further recognizes that employees’ interpretations of work events determine the emotional responses they trigger (Weiss and Cropanzano, 1996; Higgins, 2001). Positive working events, such as receiving constructive feedback or recognition, tend to evoke positive emotions, leading to increased job satisfaction, motivation, and a stronger sense of organizational commitment. These positive outcomes can enhance job performance and overall employee well-being, ultimately benefiting the organization. On the other hand, negative work events, like perceived unfairness or job insecurity, can provoke feelings of threat, dissatisfaction, and stress, which may lead to higher turnover intentions and reduced performance.

Most research has predominantly concentrated on the outcomes of negative work events, often overlooking the potential benefits of positive events on employee well-being. In the current post-pandemic environment, where employees face increased stress and uncertainty, exploring the impact of positive work events becomes crucial. Such research can provide practical guidance to organizations on how to utilize these events to enhance employees’ work performance and overall well-being.

### 2.3 Leader tolerance and work engagement

Work engagement measures the extent to which employees invest their mental, emotional, and physical resources into their job performance (Kahn, 1990). It is defined as a favorable motivational state comprising three core components: vigor, dedication, and absorption (Schaufeli et al., 2002; Bakker et al., 2014). These characteristics represent high energy and mental resilience (vigor), a strong sense of meaning and enthusiasm (dedication), and deep focus and enjoyment (absorption) that employees display in their work (Schaufeli et al., 2002). Increasingly, it is recognized as a key indicator of employee well-being and is closely linked to improved performance and adaptability within organizations (Ouweneel et al., 2011). Based on the meta-analysis performed by Björk et al. (2021), leadership plays a significant role in enhancing work engagement among employees. Positive leadership approaches, such as engaging, transformational, and servant leadership, have proven effective in this regard (De Clercq et al., 2014; Monje-Amor et al., 2020; Schaufeli, 2021).

When employees perceive that their leaders are tolerant of unintentional mistakes, such as allowing employees to correct errors without public blame, it triggers a positive work event with significant emotional implications. In high-pressure and high-risk medical environments, where errors can lead to serious consequences, leaders’ tolerant attitudes stand out and have a strong emotional impact on employees. This is due to the salience of such events in these contexts.

Drawing on AET, leader tolerance directly influences employees’ work engagement through two main paths. First, it stimulates their followers’ positive emotions to elevate work engagement. For instance, when a leader offers constructive feedback instead of punishment for a minor error, it triggers positive emotions like gratitude and relief in employees. Positive emotions can enhance employees’ well-being, which often acts as a precursor to enhanced work engagement (Avey et al., 2008; Ouweneel et al., 2012). Leader tolerance can also make employees feel trusted and respected, creating a psychologically safe atmosphere (Zhou and Cheng, 2020; Zhang et al., 2024). This sense of psychological safety encourages employees to take risks and engage in learning and development activities, which in turn enhances their work engagement (Kahn, 1990; Qasim et al., 2022).

Second, leader tolerance reduces employees’ negative emotions, which is crucial for maintaining their work engagement (Yukl, 2008). Negative emotions such as shame, anxiety, and fear of punishment often arise when employees make mistakes. These emotions can be highly distressing and can significantly impact employees’ well-being, making them feel less motivated and engaged in their work. Leaders’ tolerant behaviors can not only reduce the immediate negative emotional impact but also helps prevent long-term distress. For example, instead of publicly criticizing an employee for a minor error, a leader might offer guidance and support, helping the employee to correct the mistake without feeling ashamed or anxious. This approach not only alleviates the negative emotions associated with the mistake but also prevents the frustration that may arise from resource loss, such as time and reputation, due to errors (Hobfoll, 1989).

In high-pressure and high-risk medical environments, leader tolerance not only creates a psychologically safe atmosphere, but also fosters a learning-oriented culture. This encourages employees to engage in learning and development activities, further enhancing their work engagement.

According to Abdullah et al. (2021), work engagement has an emotional component and can be influenced by positive organizational indicators. When employees experience positive emotions due to leader tolerance, their work engagement is likely to increase. Increased positive emotions and decreased negative emotions can directly boost employees’ energy levels and focus (Sonnentag, 2003). Experimental studies have shown that positive emotions can form intellectual resources (Fredrickson, 1998), enabling employees to become more involved in their work. Additionally, a tolerant leadership style can enhance intrinsic motivation, encouraging followers to invest more energy into their tasks due to a diminished fear of failure (Zhang et al., 2024).

On the contrary, leaders who exhibit low tolerance can be perceived as a source of unfavorable events at work. For instance, employees may face penalties for their errors and lose future development opportunities. These can trigger adverse emotions such as anxiety, fear, distrust, and burnout. These negative emotions can, in turn, affect their well-being appraisals, making them feel that their well-being is compromised and leading to decreased work engagement. Moreover, the psychological insecurity resulting from these negative feelings may also reduce their job investment as they try to avoid further losses. Abdullah and Al-Abrrow (2024) demonstrated that abusive leadership, such as criticizing and blaming subordinates for their mistakes, can damage the psychological contract between leaders and their followers. This damage can lead to job stress and burnout among employees (Abdullah and Al-Abrrow, 2024), potentially reducing their investment and engagement in their work.

Thus, we propose:

*H1*: Leader tolerance positively influences employees’ work engagement.

### 2.4 Mediating role of perceived organizational support

POS captures how employees interpret the organization’s appreciation of their input and concern for their personal welfare (Eisenberger et al., 1986). Factors influencing POS include organizational justice, leadership styles, human resource management practices, and work conditions (Eisenberger et al., 2020). Leaders enhance POS by fostering internal trust and respect and by providing essential resources for employees to perform their tasks (Liu et al., 2023; Jun et al., 2023). With strong support from the organization, employees are inclined to display enhanced commitment, loyalty and enthusiasm for their work (Eisenberger et al., 2020). Additionally, a strong sense of POS is associated with fewer counterproductive work behaviors (De Clercq et al., 2021).

It is posited that leader tolerance influences employees’ work engagement through POS. As stated in Section 2.3, leader tolerance affects employees’ emotions by increasing positive emotions and reducing negative ones. According to AET, positive emotions lead employees to make positive evaluations of the organizational environment. When employees feel supported due to leader tolerance, they perceive the organization as supportive and attribute the leader’s behavior to the organization’s overall supportive policies and culture (Eisenberger et al., 1986). This strengthens their belief that the organization is committed to their well-being and development, thereby increasing their POS (Jun et al., 2023).

The relationship between POS and work engagement can be explained by the Conservation of Resources (COR) theory and Social Exchange Theory. According to COR theory, POS acts as a psychological resource that can reduce employees’ concerns about resource depletion, allowing them to invest more energy into their work (Hobfoll, 1989). Social Exchange Theory posits that employees’ perception of organizational support can lead to heightened work engagement as a reciprocal action (Blau, 1964). Empirical evidence suggests that a strong sense of POS can significantly boost work engagement, particularly in high-stress occupations like nursing (Aldabbas et al., 2023; Al-Hamdan and Issa, 2022; Mehrad et al., 2022; Wang et al., 2017).

As demonstrated by Abdullah et al. (2021), organizational support practices can create a sense of safety, leading employees to become more physically and psychologically linked to their work. POS plays a crucial role in this process by mediating the relationship between leader tolerance and work engagement. It transforms the positive emotions triggered by leader tolerance into a stronger commitment to the organization and a higher level of work engagement. Research has empirically validated the mediating role of POS between supervisor support and employee engagement (Mehrad et al., 2022). Therefore, leader tolerance, through the mediating effect of POS, can significantly enhance employees’ work engagement.

Noticeably, leaders with low tolerance levels can trigger negative emotional responses, such as anxiety, among employees, which may weaken their perception of organizational support. This reduced perception can strain the supervisor-subordinate relationship, leading employees to feel unacknowledged and deprived of career growth opportunities (Cole et al., 2006; Eisenberger et al., 2020). As POS diminishes, employees might disengage from their work, resulting in lower overall engagement. The study by Sun et al. (2022) among Chinese nurses proved that leaders’ blame on their subordinates’ mistakes can deprive their perceived support from organizations, and thus, negatively influence their work engagement.

Thus, we propose that:

*H2*: POS mediates the positive relationship between leader tolerance and employees’ work engagement.

To examine the mediating role of POS, we will employ the Bootstrap method, which provides confidence intervals to assess the significance of the indirect effect. A detailed explanation of the Bootstrap method and its implementation will be provided in Chapter 3, “Methods.”

### 2.5 Mediating role of organizational identification

Organizational identification measures the degree to which individuals incorporate their organization into their self-concept (Mael and Ashforth, 1992). Strong organizational identification indicates that employees are more inclined to embrace organizational values and norms and develop a stronger organizational affiliation (Simbula et al., 2023). Researchers have noted that it can encourage favorable work attitudes and practices, benefiting both individuals and organizations (Karanika-Murray et al., 2015; Lee et al., 2015).

Equally, leadership style and interactions with leaders profoundly affect the cultivation of employees’ affiliation with the organization (Weisman et al., 2023). For example, servant, transformational, and self-sacrificial leadership styles have been effective in fostering organizational identification by demonstrating leaders’ genuine concern for their followers’ well-being and professional growth (Weisman et al., 2023). Conversely, abusive leadership tends to diminish employees’ organizational identification and increase deviant behaviors (Liu et al., 2021).

Based on AET, leader tolerance positively influences employees’ work engagement, with organizational identification acting as a crucial mediator. When leaders demonstrate tolerance toward employees’ unintentional mistakes, it is perceived as a positive work event that generates positive emotions, for instance, gratitude, and diminishes negative emotions, such as shame and anxiety. These positive emotional reactions can stimulate affective-driven identification.

In line with Appraisal Theory (McEachrane, 2009), positive emotions lead employees to regard the organization as an extension of the “self,” thereby strengthening their identification with the organization. The study by Ko and Choi (2021) in South Korea demonstrated that positive emotion can increase employees’ organizational identification. Therefore, when employees feel respected due to leader tolerance, they are more likely to internalize organizational values. This internalization, combined with the sense of being valued by the organization, cultivates a strong psychological attachment to the organization, which is the essence of organizational identification (Allen et al., 2021).

Employees with a strong sense of organizational identification are more likely to actively engage in their work tasks (Karanika-Murray et al., 2015). Employees with high organizational identification are inclined to engage in behaviors to maintain their social identity as “organizational members” (Mael and Ashforth, 1992). They are more likely to be committed to the organization and exert extra effort to attain organizational goals. Moreover, organizational identification functions as a psychological resource that mitigates stress and promotes energy investment (Allen et al., 2016). The study conducted by Arshad et al. (2022) showed that support from leaders can boost followers’ work engagement through organizational identification. Hence, it is expected that leader tolerance can enhance employees’ identification with their organizations, and subsequently, increase their work engagement.

Conversely, leaders with low tolerance can negatively influence employees’ work engagement by diminishing their organizational identification. When leaders are intolerant, it can be perceived as a negative work event, leading to adverse emotions such as depression, guilt, and sadness (Allen et al., 2021). Such adverse emotions can reduce employees’ sense of belonging to the organization and reduce their willingness to invest effort and resources into their work. Additionally, the disrespect and distrust from intolerant leaders can create feelings of isolation, further lowering employees’ engagement with their work.

Hence, it is posited:

*H3*: Organizational identification mediates the positive relationship between leader tolerance and employees’ work engagement.

The mediation effect of organizational identification will be tested using the Bootstrap method, as mentioned in Section 2.4.

### 2.6 The chain mediating role of perceived organizational support and organizational identification

We propose a sequential mediation model where leader tolerance influences employees’ work engagement through two key pathways: POS and organizational identification. When leaders show tolerance toward their followers’ mistakes, employees view these actions as positive work events, which generate favorable emotions and restrain negative feelings. These emotions lead to an increased perception of organizational support. The positive relationship between leader tolerance and POS is evident when employees view tolerant leaders as a sign that the organization values and supports them. For instance, a leader who offers guidance instead of blame for a minor error makes employees feel highly supported.

This enhanced POS then strengthens organizational identification. Employees who feel supported by the organization see themselves as valued members and internalize its values and goals, thus increasing their identification with the organization (Edwards and Peccei, 2010). For example, a supported employee may actively participate in organizational activities and promote interests, showing a strong sense of identification.

This increased organizational identification further results in higher work engagement (Karanika-Murray et al., 2015). Staff members who have a strong sense of identification with the organization are more inclined to put their physical, cognitive, and psychological resources into their work. They take on challenging tasks, work longer hours, and are more focused and committed, all of which contribute to higher work engagement. The chain mediation process is clear: leader tolerance enhances POS, which in turn strengthens organizational identification, and finally leads to increased work engagement.

Conversely, leaders who exhibit low tolerance, for example, public criticism or blame for minor errors, it can trigger immediate negative emotional responses from employees, such as feelings or frustration, disappointment, and being undervalued. These negative emotions often lead employees to perceive a lack of organizational support. When employees feel unsupported, their sense of organizational identification is negatively affected. The relationship between POS and organizational identification is such that when POS is low, organizational identification is also likely to be weak. This weakened connection between employees and the organization can result in decreased work engagement. Employees who feel unsupported and have a weak sense of identification with the organization are less likely to be motivated to invest their physical, cognitive, and psychological resources into their work, leading to lower levels of work engagement. The chain mediation process here is that low leader tolerance leads to low POS, which in turn leads to weak organizational identification, and finally results in decreased work engagement.

Our proposed chain mediation model suggests that leader tolerance affects work engagement in two stages: first by enhancing POS and then by strengthening organizational identification. The process can be described as follows: leader tolerance → POS → organizational identification → work engagement.

Therefore, it is posited:

*H4*: POS and organizational identification sequentially mediate the positive relationship between leader tolerance and employees’ work engagement.

**Figure 1:**
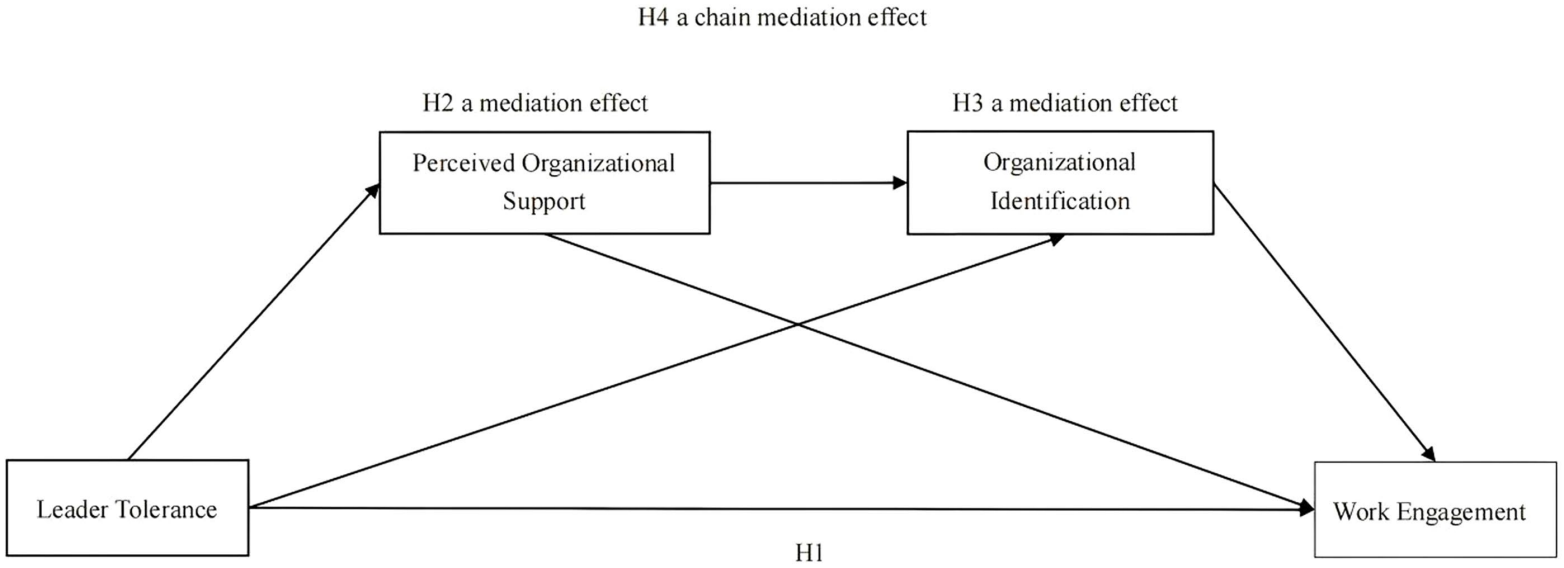
Theoretical model.

To more clearly depict the hypothesized model, Figure 1 presents a theoretical framework diagram that illustrates the relationships between the variables and indicates the corresponding hypothesis numbers. To examine the mediating roles of both POS and organizational identification, we will employ the Bootstrap method. This approach generates confidence intervals, enabling us to evaluate the significance of the indirect effects. A comprehensive explanation of the Bootstrap method and its implementation will be provided in Chapter 3, “Methods.”

## 3 Methods

### 3.1 Sample

The participants in this study were employees from a public hospital in Shandong Province, China. The hospital was selected for several reasons. First, as the largest municipal hospital in a third-tier city in the Province, it boasts a large size and comprehensive services. This translates to a diverse patient population and a wide array of medical staff roles, ensuring a comprehensive and representative sample. Second, the hospital had previously encountered issues stemming from low leader tolerance, which led to employee dissatisfaction and low work engagement. It is currently undergoing reforms to address these problems, making it an ideal setting to study the impact of leader tolerance on work engagement. Over the past few years, many hospitals in China have carried out similar management adjustments to cope with the changing environment. Lastly, the high-pressure work setting in the hospital also adds to the significance of the study. For instance, the hospital’s average number of outpatient and emergency department visits from 2023 to 2024 was approximately 1.25 million per year, which is indicative of the overall situation of hospitals in China.

In this study, we adopted random sampling. The logic behind this selection is that random sampling ensures each individual in the population has an equal likelihood of selection. This approach effectively reduces selection bias and enhances the representativeness of the sample. In a large-scale and complex setting like a hospital, where numerous factors may influence employees’ work engagement, using random sampling ensures that the sample more accurately reflects the true characteristics of the entire medical staff population. Consequently, the research findings are more likely to be generalizable to the whole hospital and even similar medical institutions.

A department manager initially contacted potential participants and invited the front-line staff to take part in an anonymous online survey. The survey introduction expressed gratitude for their participation, explained its objectives, and assured the confidentiality of their responses. We distributed 737 questionnaires to the front-line staff and received 500 responses. After conducting data cleaning, we retained 435 valid responses, achieving an effective response rate of 59.02%.

The data cleaning process involved 3 steps to ensure data quality. First, we removed incomplete responses, eliminating questionnaires that were not fully completed. Second, we excluded invalid responses. Such as those where respondents had filled in the same number for all items, as these are unlikely to provide meaningful data. Third, we corrected erroneous information, removing questionnaires with illogical personal details, such as cases where the reported age was less than the reported work tenure.

The demographic details of the participants are provided in Table 1. The average age of the respondents is 37.29 years, ranging from 21 to 61 years. Their average tenure with the organization is 12.34 years, with the longest tenure being 41 years and the shortest 1 year. The sample includes 22.30% male and 77.70% female participants. Among them, 116 are doctors, 257 are nurses, and the remaining respondents hold other front-line positions such as b-ultrasound technicians and pharmacists.

**Table 1:** Sample description.

Individual characteristics	Category	Quantity	Percentage
Gender	Male	97	22.30%
	Female	338	77.70%
Age	21–30	55	12.64%
	31–40	252	57.93%
	41–50	83	19.08%
	51–61	45	10.35%
Organizational tenure	1–10 years	233	53.56%
	11–20 years	135	31.03%
	21–30 years	34	7.82%
	32–41 years	33	7.59%
Educational background	Junior high school and below	2	0.46%
	Senior high school or equivalent	4	0.92%
	College diploma	40	9.20%
	Bachelor degree	308	70.80%
	Master degree and above	81	18.62%
Job	Doctor	116	26.67%
	Nurse	257	59.08%
	Others	62	14.25%

*N = 435.*

We compared the demographic attributes of the sample with the overall hospital population to make sure the sample was representative. The proportion of participants in our sample aligns with the overall distribution of employees in the hospital, particularly in terms of gender, education, and department. For instance, the gender distribution in our sample (22.30% male and 77.70% female) closely mirrors the gender distribution of the hospital’s front-line staff. Additionally, the sample includes a diverse range of job roles, which reflects the actual composition of the hospital’s workforce. The average age and tenure of the participants also align with the hospital’s demographic profile, ensuring that the sample is not skewed toward any particular age group or level of experience.

### 3.2 Measurement

We used a 5-point Likert scale for survey responses, where “1” represented “strongly disagree” and “5” represented “strongly agree.” All scales, except for the leader tolerance scale, were originally in English. To ensure accuracy, we adopted the method of translation and back-translation following Brislin’s (1970) guidelines.

#### 3.2.1 Leader tolerance

We used the four-item scale developed by Zhang and Tang (2016), which was culturally adapted for Chinese organizational contexts with high power distance. Sample items include “When subordinates make mistakes unintentionally, my leader tolerates their errors,” and “My leader forgives errors or failures made by his or her subordinates. Although the scale was initially validated in general enterprises, its applicability to healthcare settings was confirmed though the following steps. First, cognitive interviews with 12 medical staff (6 nurses, 4 physicians, and 2 technicians) were conducted during the pilot phase. Participants confirmed that the four items clearly reflected error tolerance dynamics in hospital workflows without needing medical jargon. Second, the scale demonstrated strong reliability (α = 0.807) in a pre-study of 157 employees from high-stress manufacturing firms with zero-tolerance management practices, a context psychologically analogous to healthcare. In the current study, the scale exhibited high internal consistency (α = 0.874).

#### 3.2.2 Perceived organizational support

POS was assessed using the six-item scale developed by Eisenberger et al. (1986). Item 5 is reverse-scored. Sample items are “The hospital values my contribution to its well-being,” “The hospital shows very little concern for me,” and “Help is available from the hospital when I have a problem.” This scale demonstrated an internal consistency coefficient (α) of 0.892 in our study.

#### 3.2.3 Organizational identification

We used a six-item scale from Mael and Ashforth (1992) to measure organizational identification. Sample items include “When someone criticizes the hospital, it feels like a personal insult,” and “The hospital’s success is my success.” In this research, the Cronbach’s α coefficient of this scale was 0.871.

#### 3.2.4 Work engagement

We assessed work engagement using the three-item short form of the Utrecht Work Engagement Scale (UWES-3, Schaufeli et al., 2019), a globally validated tool widely applied in healthcare research. Items including “At work, I feel bursting with energy,” “I am enthusiastic about my job,” and “I am immersed in my work” were retained verbatim. This can be explained as the core constructs are context-agnostic. Its applicability in the healthcare industry has been demonstrated by extensive research. For instance, For example, Muroi et al. (2023) applied the scale to Japanese nurses, and Su et al. (2023) validated its usage in the public service sector in China. The Cronbach’s α coefficient was 0.850.

#### 3.2.5 Control variables

Following previous research on work engagement, we controlled gender, age, and educational background. We coded gender as a dummy variable, with “1” for males and “2” for females. Educational background was also dummy-coded, with “1” representing Junior high school diploma and below, “2” for High school diploma or equivalent, “3” for College degree, “4” for Bachelor’s degree, “5” for Master’s degree and above.

### 3.3 Mediation effect testing

To comprehensively test the mediation effects in the relationship between leader tolerance and employees’ work engagement, we used the Bootstrap method with the SPSS Process macro (v4.1). The SPSS Process macro is a commonly utilized instrument for performing mediation, moderation, and conditional process analysis. The Bootstrap method involves repeated sampling to generate confidence intervals for the indirect effects, offering a robust assessment of the mediation effects. Specifically, we conducted the following 4 steps.

First, we specified four models. The first model had leader tolerance as the independent variable, and work engagement as the dependent variable to test the direct and total effect. The second model added POS as the mediator. The third model had leader tolerance as the independent variable, work engagement as the dependent variable, and organizational identification as the mediator. The fourth model have leader tolerance as the independent variable, work engagement as the dependent variable, and both POS and organizational identification as sequential mediators.

Second, using the Model 6 in the SPSS Process macro (v4.1), we generated 5,000 bootstrap samples to create a distribution of the indirect effects for each mediation model. Third, the SPSS Process macro (Model 6) calculated the 95% confidence intervals for the indirect effects. A confidence interval not including zero indicates a statistically significant indirect effect (MacKinnon et al., 2004). Fourth, the confidence intervals from the SPSS Process macro (Model 6) will be used to test the significance of the mediation effects of POS, organizational identification, and the chain mediation of POS and organizational identification.

By using the SPSS Process macro (Model 6) and the Bootstrap method, we provide a comprehensive and reliable assessment of the mediation effects, ensuring the robustness of our findings. This approach allows us to test the individual mediation effects of POS and organizational identification, as well as their combined effect in a chain mediation model.

### 3.4 AI-assisted processes

AI-assisted tools including Kimi (Moonshot AI, 2024) and DeepSeek-R1 (DeepSeek, 2024) were employed during the literature synthesis and data analysis phases. Researchers maintained full control over study design, result interpretation, and critical decision-making, with all AI-generated content undergoing rigorous human validation and scholarly compliance checks.

## 4 Data analysis and results

### 4.1 Test of common method biases

To address common method biases, we implemented several strategies. First, we designed the questionnaire with reverse-scored items to mitigate the impact of common method variance (CMV). We also informed participants about the study’s objectives, assuring them that their responses would be used exclusively for academic purposes and that their anonymity and confidentiality would be maintained. To further assess common method biases, we performed a Harman one-factor test. The exploratory factor analysis, conducted without rotation, revealed 4 factors with eigenvalues greater than 1. The primary factor explained 38.321% of the variance, which is below the 50% threshold (Harman, 1960). This result suggests that CMV did not significantly affect our findings.

### 4.2 Assessment of reliability and validity

We used SPSS 25.0 to evaluate the reliability of the research variables, finding that all variables had a Cronbach’s alpha coefficient (α) above 0.80, indicating acceptable reliability. The KMO values exceeded 0.70, and Bartlett’s test of sphericity was significant (*p* < 0.05). The average variance extracted (AVE) ranged from 0.766 to 0.826 with composite reliability (CR) all exceeding 0.90. The findings demonstrate good convergent validity of the scale.

The assessment of discriminant validity was based on the results of the Confirmatory Factor Analysis (CFA). CFA is a statistical technique used to verify the factor structure of a set of observed variables and is an essential tool in structural equation modeling (SEM) for evaluating the validity of a measurement model (Byrne, 2012; Kline, 2005). In CFA, the selection of appropriate model fit indices is crucial. The indices we chose—*χ*^2^/*df*, Comparative Fit Index (CFI), Tucker-Lewis Index (TLI), Root Mean Square Error of Approximation (RMSEA), and Standardized Root Mean Square Residual (SRMR)—are widely used in SEM.

In addition to using these fit indices, we employed a series of competing models to further validate the discriminant validity of the constructs. Competing models are alternative specifications of the factor structure that are hypothesized to fit the data differently. Through comparing the fit indices between the proposed model and competing models, researchers can determine whether the original model is superior in explaining the data (Kline, 2005; Byrne, 2012). CFA results of the hypothesized and 5 competing models are reported in Table 2.

**Table 2:** Confirmatory factor analysis.

Models	Factors	*χ* ^2^	*df*	χ^2^/*df*	CFI	TLI	RMSEA	SRMR
Baseline model	Four factors: LT, POS, OI, WE	456.417	146	3.126	0.905	0.889	0.070	0.062
Model 1	Three factors: LT + POS, OI, WE	942.443	149	6.325	0.757	0.721	0.111	0.104
Model 2	Three factors: LT, POS, OI + WE	900.110	149	6.041	0.770	0.736	0.108	0.118
Model 3	Two factors: LT + POS, OI + WE	1361.398	151	9.016	0.630	0.581	0.136	0.140
Model 4	Two factors: LT + POS + OI, WE	1566.583	151	10.374	0.567	0.510	0.147	0.136
Model 5	One factor: LT + POS + OI + WE	1803.777	152	11.867	0.495	0.432	0.158	0.140

*N = 435. LT, leader tolerance; POS, perceived organizational support; OI, organizational identification; WE, work engagement; χ2, chi-square; df, degree of freedom; CFI, comparative fit index; TIL, Tucker-Lewis Index; RMSEA, root mean square error of approximation; SRMR, standardized root mean square.*

Based on the index evaluation criteria suggested by Byrne (2012), the four-factor model demonstrated an acceptable fit: *χ*^2^ = 456.417, *df* = 146, *χ*^2^/*df* = 3.126, CFI = 0.905, TLI = 0.889, RMSEA = 0.070, SRMR = 0.062. The value of *χ*^2^/*df* is less than 5, which is acceptable. Although TLI is slightly lower than the acceptable criteria of 0.900, other indicators all indicate good model fit. CFI is higher than 0.900, both RMSEA and SRMR are less than 0.080 (Hu and Bentler, 1999; Schreiber et al., 2006; Steiger, 1990).

Moreover, the competing models showed inferior fit indices compared to the hypothesized model. This confirms the high discriminant validity of the constructs in the original model. The use of competing models helps to ensure that the proposed model is not only statistically sound but also theoretically justified, providing a robust validation of the factor structure.

### 4.3 Descriptive statistics and correlations

The means, standard deviations, reliabilities and correlation coefficients for the research variables are presented in Table 3. The correlation analysis indicates significant relationships between work engagement and organizational identification (*r =* 0.348*, p <* 0.01), POS (*r =* 0.517, *p* < 0.01), and leader tolerance (*r =* 0.335, *p* < 0.01). Organizational identification is positively associated with both POS (*r =* 0.360, *p <* 0.01) and leader tolerance (*r =* 0.346, *p* < 0.01). There exists a strong positive relationship between POS and leader tolerance (*r* = 0.471*, p* < 0.01). These correlations provide preliminary support for the proposed hypotheses.

**Table 3:** Descriptive statistics and analysis.

Variables	Mean	SD	1	2	3	4	5	6	7
Gender	1.780	0.417	–	−0.110^*^	−0.111^*^	−0.023	0.053	−0.090	−0.121^*^
Age	37.290	7.788	−0.110^*^	–	−0.026	−0.097^*^	0.044	0.156^**^	0.156^**^
EDU	4.060	0.594	−0.111^*^	−0.026	–	0.095^*^	0.052	0.119^*^	0.023
LT	3.626	0.738	−0.023	−0.097^*^	0.095^*^	(0.874)	0.471^**^	0.346^**^	0.335^**^
POS	3.407	0.671	0.053	0.044	0.052	0.471^**^	(0.892)	0.360^**^	0.517^**^
OI	4.067	0.625	−0.090	0.156^**^	0.119^*^	0.346^**^	0.360^**^	(0.871)	0.348^**^
WE	3.516	0.793	−0.121^*^	0.156^**^	0.023	0.335^**^	0.517^**^	0.348^**^	(0.850)

*N* = 435. ^*^p < 0.05, ^**^p < 0.01, (2-tailed). SD, standard deviation; EDU, educational background; LT, leader tolerance; POS, perceived organizational support; OI, organizational identification; WE, work engagement. For gender, 1 = male, 2 = female. For educational background, 1 = junior high school and below, 2 = high school or equivalent, 3 = College diploma, 4 = Bachelor degree, 5 = Master degree and above. Cronbach’s alphas are shown in in the parentheses on the diagonal.

In addition to the main research findings related to the hypotheses, several other important correlations were observed. The negative correlation between female gender and work engagement (*r =* −0.121*, p* < 0.05) may reflect women’s overrepresentation in high-burnout clinical roles and greater familial care responsibilities in China’s healthcare sector (Yuan et al., 2024). The positive education-organizational identification link (*r =* 0.119, *p* < 0.05) may reflect higher-educated employees’ stronger mission alignment. Age is observed negatively correlated with leader tolerance (*r =* −0.097, *p <* 0.05) but positively with work engagement (*r =* 0.156, *p* < 0.01). This may be due to the fact that leaders are more tolerant of errors made by younger employees than those made by older employees. Besides, older employees may have a clearer understanding of their career goals and responsibilities, and a better balance between work and life, thus showing higher work engagement.

### 4.4 Hypothesis testing

For this study, the Process macro v4.1 in SPSS was utilized to test the sequential mediation model. Applying a bootstrapping method with 5,000 resamples and a 95% confidence level, we assessed the mediating roles of POS and organizational identification while controlling for age, gender and educational background. As shown in Table 4, leader tolerance has a positive and significant effect on work engagement (β = 0.377, *p* < 0.001), confirming Hypothesis 1. This effect size indicates a moderately strong relationship between leader tolerance and work engagement. It also significantly predicts POS (β = 0.437, *p* < 0.001) and organizational identification (β = 0.203, *p <* 0.001), suggesting that leader tolerance has a substantial impact on there variables. POS significantly impacts organizational identification (β = 0.223, *p* < 0.001). Moreover, both POS (β = 0.501, *p* < 0.001) and organizational identification (β = 0.174, *p <* 0.05) positively predict work engagement. Even after accounting for these mediating effects, the link between leader tolerance and work engagement remains significant (β = 0.106, *p* < 0.05), indicating that POS and organizational identification partially mediate the impact of leader tolerance on work engagement. The practical significance of these findings is notable, as the effect sizes suggest that leader tolerance has a meaningful impact on work engagement, POS, and organizational identification.

**Table 4:** Regression analysis.

Outcome variable	Predictor	*R*	*R* ^2^	*F*-value	*β*	*t*-value
WE		0.396	0.157	19.964^***^		
	Gender				−0.181	−2.115^*^
	Age				0.018	3.995^***^
	EDU				−0.021	−0.359
	LT				0.377	7.862^***^
POS		0.485	0.236	33.142^***^		
	Gender				0.124	1.806
	Age				0.009	2.326^*^
	EDU				0.020	0.414
	LT				0.437	11.292^***^
OI		0.459	0.211	22.907^***^		
	Gender				−0.105	−1.605
	Age				0.013	3.747^***^
	EDU				0.084	1.848
	LT				0.203	4.864^***^
	POS				0.223	4.886^***^
WE		0.575	0.331	35.297^***^		
	Gender				−0.229	−2.988^*^
	Age				0.011	2.718^*^
	EDU				−0.047	−0.876
	LT				0.106	2.120^*^
	POS				0.501	9.121^***^
	OI				0.174	3.091^*^

*N* = 435. ^*^p < 0.05, ^**^p < 0.01, ^***^p < 0.001, (2-tailed). EDU, educational background; LT, leader tolerance; POS, perceived organizational support; OI, organizational identification; WE, work engagement. For gender, 1 = male, 2 = female. For educational background, 1 = junior high school and below, 2 = high school or equivalent, 3 = College diploma, 4 = Bachelor degree, 5 = Master degree and above.

We conducted additional validation of the hypothesized mediation effects using bootstrapping, as detailed in Table 5. The results confirm significant mediating effects of POS and organizational identification. The total effect of leader tolerance on work engagement is 0.377, with a direct effect of 0.106. Likewise, the total mediating effect is 0.271, which can be divided into three distinct pathways. First, the indirect effect of leader tolerance on their followers’ work engagement through POS is 0.219, with a 95% confidence interval (CI) of [0.156, 0.290], which does not include zero, thus supporting Hypothesis 2. Furthermore, the second mediation path, from leader tolerance through organizational identification to work engagement, shows an indirect effect of 0.035 (95% CI [0.009, 0.071]), supporting Hypothesis 3. Equally, the sequential mediation effect from leader tolerance through both POS and organizational identification to work engagement is 0.017, with a 95% CI of [0.005, 0.033], confirming Hypothesis 4 of the chain mediation effect.

**Table 5:** Analysis of the mediating effect of POS and OI.

	Effect	BootSE	95% LLCI	95% ULCI
Total effect	0.377	0.048	0.283	0.472
Direct effect	0.106	0.050	0.008	0.205
Total indirect effect	0.271	0.038	0.200	0.352
Path 1: LT → POS → WE	0.219	0.034	0.156	0.290
Path 2: LT → OI → WE	0.035	0.016	0.009	0.071
Path 3: LT → POS → OI → WE	0.017	0.007	0.005	0.033

*N = 435. Bootstrap n = 5,000. LT, leader tolerance; POS, perceived organizational support; OI, organizational identification; WE, work engagement; LLCI, lower level confidence interval; ULCI, upper level confidence interval.*

## 5 Discussion

This study sought to explore how leader tolerance affects employees’ work engagement, focusing specifically on the mediating roles of POS and organizational identification. Our research provides an in-depth understanding of the link between leaders’ behaviors and the engagement of their subordinates.

Our first hypothesis, which proposed that leader tolerance positively impacts employees’ work engagement, received strong empirical support. This finding aligns with AET, which suggests that leaders’ tolerance for employees’ mistakes is perceived as a positive work event. When leaders show tolerance, they create a sense of psychological safety and reduce employees’ stress, making them feel more secure and valued. This recognition of effort, even when mistakes occur, motivates employees to engage more with their work. The findings confirm previous research that highlights how leader tolerance fosters proactive work behaviors through psychological safety (Zhou and Cheng, 2020) and strengthens employees’ mental resilience (Zhang et al., 2024). The findings also reinforce the idea that supportive leadership, which includes tolerance for mistakes, promotes a more engaged and committed workforce.

Our second hypothesis examined whether POS mediates the association between leader tolerance and employees’ work engagement. The empirical analysis indicates significant mediation, showing that employees who experience greater tolerance from their leaders also perceive higher organizational support. Hence, this enhances the sense of support and also increases work engagement. This finding emphasizes the crucial role of POS as a key mechanism linking leadership behaviors to employees’ attitudes and work-related behaviors.

Contemporaneously, organizational identification emerged as a significant mediator in our study. The results supported the hypothesis that leader tolerance enhances employees’ organizational identification, which subsequently boost their work engagement. This finding builds on Cao et al. (2024), who put forward that leader tolerance boosts employees’ psychological ownership, which can develop into organizational identification. Our research underscores the importance of organizational identification as a vital psychological process linking leadership practices to employee engagement.

The final hypothesis, which proposed a chain mediation model involving POS and organizational identification as sequential mediators between leader tolerance and work engagement, was also verified. The results demonstrate that leader tolerance influences work engagement through the serial pathways of POS and organizational identification. This finding highlight that the effects of leadership behaviors on employees’ work attitudes are not merely direct but are mediated through a series of psychological states, such as POS and organizational identification. This complexity emphasizes the nuanced association between leadership practices and employee engagement.

### 5.1 Theoretical contributions

This study makes several significant theoretical contributions that address the gaps in prior research and provide new insights into the emotional responses to leadership behaviors and their effects on work engagement.

First, this study delves into the emotional impact of leader tolerance through the lens of AET, filling a crucial gap in the literature. Previous studies using AET have largely focused on negative work events such as negative performance feedback (Alam and Singh, 2021), leader narcissism (Chen et al., 2020), and abusive supervision (Tillman et al., 2018). While these studies have provided valuable insights into the negative emotional impacts of leadership behaviors, they have largely overlooked the positive emotional effects of leader tolerance. By highlighting the positive emotions generated by leader tolerance, this study offers a new perspective on the affective dynamics of leader-follower relationships and their influence on work engagement. It expands the application of AET to positive emotional responses in the workplace, complementing the existing literature that has primarily focused on negative events and emotions. The research results echo the suggestion by Abdullah et al. (2021) that work engagement has emotional components, emphasizing the need for managers to pay attention to their subordinates’ emotional experiences.

Second, the study introduces POS as a mediating variable, providing a broader perspective than previous research that focused on individual psychology. POS reflects employees’ perception of the organization’s resource allocation and other supportive actions. While prior studies have explored the role of mediating variables such as psychological ownership and moral disengagement in leader tolerance, these variables mainly center on employees’ individual psychological states or behavioral inclinations, with a lack of systematic investigation into organizational-level support. Our research incorporates POS as a mediating variable, underscoring that leader tolerance not only influences employees’ individual psychological states but also amplifies their work engagement by elevating their overall perception of support from the organization. Compared to previous mediators that focused on individual psychology and behavior, POS offers a more comprehensive understanding of the impact of leader tolerance on employee behavior, revealing how leader tolerance can have an impact beyond the individual level through the lens of employees’ perception of organizational support.

Third, this research investigates the impact of leader tolerance on employees’ work engagement, which is directly related to job performance. While prior research has mainly focused on how leader tolerance affects employees’ psychological states, such as psychological resilience and psychological safety, paying little attention to its influence on job performance. This study addresses this gap by considering work engagement as the final outcome variable and exploring the mechanism through which leader tolerance affects work engagement via the mediating effects of POS and organizational identification. This comprehensive exploration not only compensates for the shortcomings of previous research but also provides a new perspective on the antecedents of work engagement, enabling organizations to better utilize leadership behaviors to enhance employees’ work engagement and improve organizational performance.

Finally, by incorporating AET theory along with psychological safety and learning - oriented leadership to explain the influence of tolerant leadership, this study expands the theoretical framework of inclusive leadership. Inclusive leadership is characterized by openness, acceptance, and support for diverse perspectives and ideas. Leader tolerance is an important aspect of inclusive leadership, as it demonstrates the leader’s willingness to accept and support employees’ mistakes and learning processes. This study highlights the importance of leader tolerance in fostering psychological safety and a learning-oriented culture, which are crucial for enhancing employee engagement and performance. By emphasizing the emotional mechanisms through which leader tolerance influences employees’ attitudes and behaviors, this study provides a deeper understanding of the role of emotions in inclusive leadership and how it can positively impact employees. This, in turn, enriches the theoretical framework of inclusive leadership and provides new insights into how leaders can create a more supportive and engaging work environment.

In terms of challenging the traditional Leader-Member Exchange (LMX) theory, this study emphasizes the emotional mechanisms of leader tolerance. LMX theory focuses on the cognitive aspects of the leader - member relationship, such as resource exchange and trust. However, this study highlights the importance of emotions in the leader-follower relationship. By showing that leader tolerance can generate positive emotions among employees, which in turn enhance their work engagement. This study also challenges the assumption of differentiated relationships in LMX theory. It demonstrates that leader tolerance can positively impact all followers, establishing high-quality relationships and questioning the difficulty of achieving consensus in traditional LMX theory.

### 5.2 Practical implications

In high-pressure fields like public health, leaders are pivotal in boosting employees’ work engagement and psychological safety. To better utilize the research findings and improve work engagement in such demanding environments, this study offers the following practical advice for leaders.

First, leaders should cultivate a growth-oriented mindset among employees. This means that mistakes are seen as opportunities for learning and development rather than as signs of failure. For example, when a public health campaign to promote healthy eating habits does not achieve the expected results, the leader can guide the team to analyze the reasons from a psychological perspective. It could be that the messages were not effectively communicated to the target audience, or the incentives offered were not attractive enough. By focusing on these psychological factors and adjusting the strategies accordingly, employees will feel more supported and their sense of organizational identification will be enhanced, leading to increased work engagement.

Second, leaders in high-pressure environments should engage in transparent communication with their team members. For instance, in a public health organization dealing with an infectious disease outbreak, the leader can regularly hold team meetings to share the latest information about the situation, including the challenges and the uncertainties. At the same time, the leader should encourage team members to express their concerns and ideas freely. When an employee makes a mistake, such as a wrong prediction about the spread of the disease, the leader can use this as an opportunity to emphasize that it’s normal to have different opinions and that everyone’s input is valued. This kind of transparent communication can greatly enhance psychological safety and make employees more willing to engage in their work.

Third, in high-pressure work settings, leaders should provide psychological support and resources to help employees cope with the stress caused by mistakes. For example, a public health organization can offer access to professional counseling services for employees who are feeling overwhelmed after a project failure. The leader can also organize stress-management workshops to teach employees techniques such as mindfulness and relaxation exercises. These psychological support measures can help employees recover from the negative emotions caused by mistakes and improve their work engagement.

Fourth, leaders should provide timely and constructive feedback to employees. In high-pressure environments like public health, employees often face complex and challenging tasks. Leaders should not only point out mistakes but also offer specific suggestions for improvement. For example, when an employee makes an error in data analysis during a public health research project, the leader can provide detailed feedback on the correct analytical methods and tools, and guide the employee to re-analyze the data. This kind of timely and constructive feedback can help employees quickly correct their mistakes and improve their work skills, which in turn enhances their work engagement.

The final recommendation is leaders should encourage team collaboration and knowledge sharing. In public health work, different team members may have different areas of expertise and experience. Leaders can organize regular team meetings or knowledge-sharing sessions to encourage employees to share their work experience and insights. For example, after a successful public health intervention, the leader can ask team members to share their experiences and lessons learned in the project, so that other members can learn from them. This kind of team collaboration and knowledge sharing can not only improve the overall work efficiency of the team but also enhance employees’ sense of organizational identification and work engagement.

## 6 Limitations and future research directions

Despite the theoretical and practical contributions, this research has limitations that warrant consideration. First, the sample comprises hospital staff, whose experiences may differ from those of employees in other industries due to unique management systems and organizational structures. This specificity may constrain the applicability of the results across different context. Given the ongoing organizational restructuring and digital transformation across various sectors, which can induce job insecurity, the roles that tolerant leaders can play may differ. Future research should include diverse samples to validate the observed relationships, thereby enhancing the validity of the findings and providing a broader understanding of leader tolerance in various contexts. Additionally, it should be noted that all participants in this study are Chinese. Given that Chinese culture is characterized by high power distance and collectivism, it is recommended that future research can be conducted in countries with different cultural backgrounds. This will help to verify the contextual boundaries of the research findings and facilitate cross-cultural comparisons.

Second, the reliance on self-reported data collected at a single time point may constrain the ability to make causal inferences. Although we considered conducting the survey across different shifts to mitigate common method biases, this approach was not feasible due to the high workload of medical staff. Efforts to control for common method biases included using reverse-scored questions, and the Harmon one-factor test suggested minimal bias. However, this does not entirely eliminate the potential influence of common method biases. Future studies should utilize multi-source and multi-wave surveys to strengthen result validity. Engaging both leaders and followers in surveys could provide a more comprehensive dataset. Additionally, incorporating techniques such as response surface analysis and multilevel regression could enrich the research outcome by accounting for variability and complexity.

Third, this study concentrated on a chained mediation model, highlighting opportunities to explore contextual variables in leader tolerance research. Based on AET (Weiss and Cropanzano, 1996), individual traits might moderate how work events affect employees’ emotional responses. Future research can examine how individual traits, such as age and gender, moderate the relationship between leader tolerance and employees’ reactions. While previous research has noted the cross-level moderation effect of team initiative climate (Zhou and Cheng, 2020), other potential organizational and occupational moderators remain under-explored. For example, strict organizational policies may limit the impact of leader tolerance on employee behavior, while more flexible policies may enhance this impact. Similarly, different job roles may have different expectations and responsibilities, which may moderate the relationship between leader tolerance and employee behavior. Future studies can also address these limitations by investigating the moderating roles of organizational contexts, and job characteristics. Identifying these factors will help organizations create more targeted and effective error management strategies.

## 7 Conclusion

In conclusion, this research underscores the significant impact of leader tolerance on employee work engagement, highlighting its role as both a direct influence and a mediator through POS and organizational identification. By demonstrating that leader tolerance enhances employees’ affective experiences and engagement, our research provides valuable insights into how positive leadership behaviors can foster a more engaged and resilient workforce. This study advances theoretical understanding by applying AET to positive work events and offers practical implications for developing supportive error management strategies and leadership training programs. Future research should explore additional contextual and individual moderators to further refine strategies for enhancing employee engagement and organizational effectiveness.

Based on this study, organizations should focus on fostering a leadership culture that emphasizes tolerance and support to enhance employee engagement and resilience. Although the study provides valuable insights, it has limitations, including the focus on hospital staff, which may not generalize to other industries, and the reliance on self-rated data at a single point in time. Future studies should address these limitations by using diverse samples from various industries and utilizing multi-source, longitudinal data to better understand the dynamics of leader tolerance. Additionally, investigating potential contextual and individual moderators could provide a deeper understanding of how leader tolerance influences employee attitudes and behaviors across different settings and circumstances.

## Data Availability

The raw data supporting the conclusions of this article will be made available by the authors, without undue reservation.

## References

[ref1] Abdullah H. O. Al-Abrrow H. (2024). Decoding workplace dynamics: unveiling perceptual and attitudinal drivers of counterproductive work behaviour using hybrid SEMANN approach. Int. J. Org. Anal. 33, 479–501. doi: 10.1108/IJOA-10-2023-4019, PMID: 35579975

[ref2] Abdullah H. O. Ismail I. Alnoor A. Yaqoub E. (2021). Effect of perceived support on employee's voice behaviour through the work engagement: a moderator role of locus of control. Int. J. Proc. Manag. Benc. 11, 60–79. doi: 10.1504/IJPMB.2021.112253, PMID: 35009967

[ref3] Alam M. Singh P. (2021). Performance feedback interviews as affective events: an exploration of the impact of emotion regulation of negative performance feedback on supervisor–employee dyads. Hum. Resour. Manage. Rev. 31, 100740–100714. doi: 10.1016/j.hrmr.2019.100740, PMID: 39983460

[ref4] Aldabbas H. Pinnington A. Lahrech A. (2023). The influence of perceived organizational support on employee creativity: the mediating role of work engagement. Curr. Psychol. 42, 6501–6515. doi: 10.1007/s12144-021-01992-1

[ref5] Al-Hamdan Z. Issa H. B. (2022). The role of organizational support and self-efficacy on work engagement among registered nurses in Jordan: a descriptive study. J. Nurs. Manage. 30, 2154–2164. doi: 10.1111/jonm.13456, PMID: 34415087

[ref9008] Allen J. A. Crowe J. Baran B. E. Scott C. (2016). Organizational identification: A context‐specific mitigating resource of work–family conflict. J. Conting. Crisis Man. 24, 27–35. doi: 10.1111/1468-5973.12102

[ref6] Allen K. A. Kern M. L. Rozek C. S. Mclnerney D. M. Slavich G. M. (2021). Belonging: a review of conceptual issues, an integrative framework, and directions for future research. Austr. J. Psychol. 73, 87–102. doi: 10.1080/00049530.2021.1883409, PMID: 33958811 PMC8095671

[ref7] Arshad M. Qasim N. Farooq O. Rice J. (2022). Empowering leadership and employees' work engagement: a social identity theory perspective. Manag. Dec. 60, 1218–1236. doi: 10.1108/MD-11-2020-1485, PMID: 35579975

[ref8] Ashkanasy N. M. Dorris A. D. (2017). Emotions in the workplace. Annu. Rev. Org. Psychol. Org. Behav. 4, 67–90. doi: 10.1146/annurev-orgpsych-032516-113231

[ref9] Avey J. B. Wernsing T. S. Luthans F. (2008). Can positive employees help positive organizational change? Impact of psychological capital and emotions on relevant attitudes and behaviors. J. Appl. Behav. Sci. 44, 48–70. doi: 10.1177/0021886307311470

[ref10] Bakker A. B. Demerouti E. Sanz-Vergel A. I. (2014). Burnout and work engagement: the JD–R approach. Annu. Rev. Organ. Psychol. Organ. Behav. 1, 389–411. doi: 10.1146/annurev-orgpsych-031413-091235

[ref11] Bass B. M. Avolio B. J. (1990). Developing transformational leadership: 1992 and beyond. J. Eur. Ind. Train. 14, 21–27. doi: 10.1108/03090599010135122, PMID: 35579975

[ref12] Bell B. S. Kozlowski S. W. J. (2008). Active learning: effects of core training design elements on self-regulatory processes, learning, and adaptability. J. Appl. Psychol. 93, 296–316. doi: 10.1037/0021-9010.93.2.296, PMID: 18361633

[ref13] Björk J. M. Bolander P. Forsman A. K. (2021). Bottom-up interventions effective in promoting work engagement: a systematic review and meta-analysis. Front. Psychol. 12:730421. doi: 10.3389/fpsyg.2021.730421, PMID: 34566819 PMC8456101

[ref14] Blau P. M. (1964). Exchange and power in social life. New York: Wiley.

[ref15] Bledow R. Schmitt A. Frese M. Kühnel J. (2011). The affective shift model of work engagement. J. Appl. Psychol. 96, 1246–1257. doi: 10.1037/a0024532, PMID: 21766997

[ref16] Brief A. P. Weiss H. M. (2002). Organizational behavior: affect in the workplace. Annu. Rev. Psychol. 53, 279–307. doi: 10.1146/annurev.psych.53.100901.135156, PMID: 11752487

[ref17] Brislin R. W. (1970). Back-translation for cross-cultural research. J. Cross-Cult. Psychol. 1, 185–216. doi: 10.1177/135910457000100301

[ref18] Byrne B. M. (2012). Structural equation modeling with mplus: Basic concepts, applications, and programming. New York: Taylor and Francis.

[ref19] Cao S. Zhang H. Chen Q. (2024). Leader fault tolerance and employees’ green silent behavior: the mediating role of psychological ownership and moral disengagement. Sustain. For. 16, 1–17. doi: 10.3390/su16156431, PMID: 39857420

[ref20] Carmeli A. Reiter-Palmon R. Ziv E. (2010). Inclusive leadership and employee involvement in creative tasks in the workplace: the mediating role of psychological safety. Creat. Res. J. 22, 250–260. doi: 10.1080/10400419.2010.504654

[ref21] Chen J. T. Cheng Z. H. Wang H. Q. Li D. (2020). Does leader narcissism hinder employees taking charge? An affective events theory perspective. Soc. Behav. Pers. Int. J. 48, 1–13. doi: 10.2224/sbp.9377

[ref22] Chen Y. Wei J. Zhang J. Li X. (2021). Effect mechanism of error management climate on innovation behavior: an investigation from Chinese entrepreneurs. Front. Psychol. 12, 1–13. doi: 10.3389/fpsyg.2021.733741, PMID: 34950082 PMC8688954

[ref23] Cole M. S. Bruch H. Vogel B. (2006). Emotion as mediators of the relations between perceived supervisor support and psychological hardiness on employee cynicism. J. Org. Behav. 27, 463–484. doi: 10.1002/job.381

[ref24] De Clercq D. Bouckenooghe D. Raja U. Matsyborska G. (2014). Servant leadership and work engagement: the contingency effects of leader–follower social capital. Hum. Resour. Dev. Q. 25, 183–212. doi: 10.1002/hrdq.21185

[ref25] De Clercq D. Kundi Y. M. Sardar S. Shahid S. (2021). Perceived organizational injustice and counterproductive work behaviors: mediated by organizational identification, moderated by discretionary human resource practices. Pers. Rev. 50, 1545–1565. doi: 10.1108/PR-06-2020-0469

[ref26] Dimitrova N. G. Van Hooft E. A. J. (2021). In the eye of the beholder: leader error orientation, employee perception of leader, and employee work-related outcomes. Acad. Manag. Discov. 7, 530–553. doi: 10.5465/amd.2019.0184

[ref27] Edwards M. Peccei R. (2010). Perceived organizational support, organizational identification, and employee outcomes: testing a simultaneous multifoci model. J. Pers. Psychol. 9, 17–26. doi: 10.1027/1866-5888/a000007

[ref28] Eisenberger R. Huntington R. Hutchison S. Debora S. (1986). Perceived organizational support. J. Appl. Psychol. 71, 500–507. doi: 10.1037/0021-9010.71.3.500

[ref29] Eisenberger R. Shanock R. L. Wen X. (2020). Perceived organizational support: why caring about employees counts. Annu. Rev. Organ. Psychol. 7, 101–124. doi: 10.1146/annurev-orgpsych-012119-044917

[ref30] Eva N. Robin M. Sendjaya S. Van Dierendonck D. Liden R. C. (2019). Servant leadership: a systematic review and call for future research. Leadership Q. 30, 111–132. doi: 10.1016/j.leaqua.2018.07.004

[ref31] Fisher C. D. (2000). Mood and emotions while working: missing pieces of job satisfaction? J. Org. Behav. 21, 185–202. doi: 10.1002/(SICI)1099-1379(200003)21:2<185::AID-JOB34>3.0.CO;2-M

[ref32] Fredrickson B. L. (1998). What good are positive emotions?.Rev. Gen. Psychol. 2, 300–319. doi: 10.1037/1089-2680.2.3.300, PMID: 21850154 PMC3156001

[ref33] Frese M. Keith N. (2015). Action errors, error management, and learning in organizations. Annu. Rev. Psychol. 66, 661–687. doi: 10.1146/annurev-psych-010814-015205 25251490

[ref34] Harman H. (1960). Modern factor analysis. Chicago: University of Chicago Press.

[ref35] Higgins E. T. (2001). “Promotion and prevention experiences: relating emotions to non-emotional motivational states” in The handbook of affect and social cognition. ed. Forgas J. P. (Manwah: Lawrence Erlbaum Associates), 186–221.

[ref36] Hobfoll S. E. (1989). Conservation of resources: a new attempt at conceptualizing stress. Am. Psychol. 44, 513–524. doi: 10.1037/0003-066X.44.3.513, PMID: 2648906

[ref37] Hofmann D. A. Frese M. (2011). “Errors, error taxonomies, error prevention, and error management: laying the groundwork for discussing errors in organizations” in Error in organizations. eds. Hofmann D. A. Frese M. (New York: Routlege), 1–43.

[ref38] Hu L. T. Bentler P. M. (1999). Cutoff criteria for fit indexes in covariance structure analysis: conventional criteria versus new alternatives. Struct. Equ. Modeling 6, 1–55. doi: 10.1080/10705519909540118

[ref39] Jun K. Hu Z. Sun Y. (2023). Impact of authentic leadership on employee turnover intention: perceived supervisor support as mediator and organizational identification as moderator. Front. Psychol. 14, 1–15. doi: 10.3389/fpsyg.2023.1009639, PMID: 36760446 PMC9902360

[ref40] Kahn W. A. (1990). Psychological conditions of personal engagement and disengagement at work. Acad. Manag. J. 33, 692–724. doi: 10.2307/256287

[ref41] Karanika-Murray M. Duncan N. Pontes H. Griffiths M. (2015). Organizational identification, work engagement, and job satisfaction. J. Manag. Psychol. 30, 1019–1033. doi: 10.1108/JMP-11-2013-0359

[ref42] Kline R. B. (2005). Principles and practice of structural equation modeling. 2nd Edn. New York: Guilford Press.

[ref43] Ko S. H. Choi Y. (2021). Positive leadership and organizational identification: mediating roles of positive emotion and compassion. Prob. Pers. Manage. 19, 13–23. doi: 10.21511/ppm.19(1).2021.02, PMID: 39972643

[ref44] Lee E. S. Park T. Y. Koo B. (2015). Identifying organizational identification as a basis for attitudes and behaviors: a meta-analytic review. Psychol. Bull. 141, 1049–1080. doi: 10.1037/bul0000012, PMID: 25984729

[ref45] Liu C. Yang J. Liu J. Zhu L. (2021). The effect of abusive supervision on employee deviant behaviors: an identity-based perspective. Int. J. Hum. Resour. Manag. 32, 948–978. doi: 10.1080/09585192.2018.1511613

[ref46] Liu N. Zhang H. Zhou J. (2023). Unraveling the effect of differential leadership on employee performance: evidence from China. Front. Psychol. 14:1081073. doi: 10.3389/fpsyg.2023.1081073, PMID: 36935973 PMC10017960

[ref47] MacKinnon D. P. Lockwood C. M. Williams J. (2004). Confidence limits for the indirect effect: distribution of the product and resampling methods. Multivar. Behav. Res. 39, 99–128. doi: 10.1207/s15327906mbr3901_4, PMID: 20157642 PMC2821115

[ref48] Mael F. Ashforth B. E. (1992). Alumni and their alma mater: a partial test of the reformulated model of organizational identification. J. Org. Behav. 13, 103–123. doi: 10.1002/job.4030130202

[ref49] McEachrane M. (2009). Emotion, meaning, and appraisal theory. Theor. Psychol. 19, 33–53. doi: 10.1177/0959354308101418

[ref50] Mehrad A. Fernández-Castro J. Gómez de Olmedo M. P. G. García-Sierra R. (2022). Mediation role of perceived organizational support on Nurses' work engagement and leadership styles. Nurs. Med. J. Nurs. 12, 208–222. doi: 10.14710/nmjn.v12i2.45872

[ref51] Monje-Amor A. Vázquez J. P. A. Faíña J. A. (2020). Transformational leadership and work engagement: exploring the mediating role of structural empowerment. Eur. Manage. J. 38, 169–178. doi: 10.1016/j.emj.2019.06.007

[ref52] Muroi K. Ishitsuka M. Hachisuka T. Shibata I. Ikeda T. Hori D.. (2023). Factors associated with work engagement of nurses during the fifth wave of the COVID-19 pandemic in Japan:web based cross-sectional study. JMIR Form Res. 7:e45830. doi: 10.2196/45830, PMID: 37921864 PMC10656660

[ref53] Ouweneel E. Le Blanc P. M. Schaufeli W. B. (2011). Flourishing students: a longitudinal study on positive emotions, personal resources, and study engagement. J. Posit. Psychol. 6, 142–153. doi: 10.1080/17439760.2011.558847

[ref7001] Ouweneel E. Le Blanc P. M. Schaufeli W. B. van Wijhe C. I. (2012). Good morning, good day: A diary study on positive emotions, hope, and work engagement. Hum. Relat. 65, 1129–1154. doi: 10.1177/0018726711429382

[ref54] Oyserman D. Lee S. W. S. (2008). Does culture influence what and how we think? Effects of priming individualism and collectivism. Psychol. Bull. 134, 311–342. doi: 10.1037/0033-2909.134.2.311, PMID: 18298274

[ref55] Qasim S. Usman M. Ghani U. Khan K. (2022). Inclusive leadership and employees’ helping behaviors: role of psychological factors. Front. Psychol. 13, 1–9. doi: 10.3389/fpsyg.2022.888094, PMID: 35874383 PMC9301301

[ref56] Reason J. (1995). A systems approach to organizational error. Ergonomics 38, 1708–1721. doi: 10.1080/00140139508925221

[ref57] Schaufeli W. (2021). Engaging leadership: how to promote work engagement? Front. Psychol. 12:754556. doi: 10.3389/fpsyg.2021.754556, PMID: 34777155 PMC8578900

[ref58] Schaufeli W. B. Salanova M. González-Romá V. Bakker A. B. (2002). The measurement of engagement and burnout: a two sample confirmatory factor analytic approach. J. Happiness Stud. 3, 71–92. doi: 10.1023/A:1015630930326

[ref59] Schaufeli W. B. Shimazu A. Hakanen J. Salanova M. De Witte H. (2019). An ultra-short measure for work engagement. Eur. J. Psychol. Assess. 35, 577–591. doi: 10.1027/1015-5759/a000430

[ref60] Schreiber J. B. Nora A. F. K. Stage F. K. Barlow E. A. King J. (2006). Reporting structural equation modeling and confirmatory factor analysis results: a review. J. Educ. Res. 99, 323–338. doi: 10.3200/JOER.99.6.323-338, PMID: 25898369

[ref61] Shore L. M. Chung B. G. (2022). Inclusive leadership: how leaders sustain or discourage work group inclusion. Group Organ. Manage. 47, 723–754. doi: 10.1177/1059601121999580

[ref62] Simbula S. Margheritti S. Avanzi L. (2023). Building work engagement in organizations: a longitudinal study combining social exchange and social identity theories. Behav. Sci. 13, 1–15. doi: 10.3390/bs13020083, PMID: 36829312 PMC9952149

[ref63] Sonnentag S. (2003). Recovery, work engagement, and proactive behavior: a new look at the interface between non-work and work. J. Appl. Psychol. 88, 518–528. doi: 10.1037/0021-9010.88.3.518, PMID: 12814299

[ref64] Steiger J. H. (1990). Structural model evaluation and modification: an interval estimation approach. Behav. Res. Ther. 25, 173–180. doi: 10.1207/s15327906mbr2502_4, PMID: 26794479

[ref65] Su X. Wong V. Yip C. (2023). Validation of the ultra-short scale for measuring work engagement among social workers in Chinese contexts. Int. J. Soc. Welf. 32, 241–255. doi: 10.1111/ijsw.12552

[ref66] Sun N. Zheng Q. Li L. Zhu H. Liu X. Zhou S.. (2022). A model of abusive supervision, self-efficacy and work engagement among Chinese registered nurses: the mediating role of self-efficacy. Front. Psychol. 13:962403. doi: 10.3389/fpsyg.2022.962403, PMID: 36524172 PMC9744932

[ref67] Tang N. Jiang Y. Chen C. Zhou Z. Z. Chen C. C. Yu Z. (2015). Inclusion and inclusion management in the Chinese context: an exploratory study. Int. J. Hum. Res. Manage. 26, 856–874. doi: 10.1080/09585192.2014.985326, PMID: 39981287

[ref68] Tillman C. J. Gonzalez K. Crawford W. S. Lawrence E. R. (2018). Affective responses to abuse in the workplace: the role of hope and affective commitment. Int. J. Select. Assess. 26, 57–65. doi: 10.1111/ijsa.12203

[ref69] Van Dyck C. Dimitrova N. G. De Korne D. F. Hiddema F. (2013). “Walk the talk: leaders’ enacted priority of safety, incident reporting, and error management in improving safety, satisfaction and financial performance” in Leading in health care organizations. eds. Simons T. Leroy H. Savage G. T. (Leeds: Emerald Group Publishing Limited), 95–117. 10.1108/s1474-8231(2013)0000014009 24772884

[ref70] Wallo A. Lundqvist D. Coetzer A. (2024). Learning-oriented leadership in organizations: an integrative review of qualitative studies. Hum. Resour. Dev. Rev. 23, 230–275. doi: 10.1177/15344843241239723

[ref71] Wang X. Liu L. Zou F. Hao J. Wu H. (2017). Associations of occupational stressors, perceived organizational support, and psychological capital with work engagement among Chinese female nurses. Biomed. Res. Int. 2017:5284628. doi: 10.1155/2017/5284628, PMID: 28168198 PMC5266809

[ref72] Weisman H. Wu C. H. Yoshikawa K. Lee H. J. (2023). Antecedents of organizational identification: a review and agenda for future research. J. Manage. 49, 2030–2061. doi: 10.1177/01492063221140049, PMID: 39981163

[ref73] Weiss H. M. Cropanzano R. (1996). (1996). Affective events theory. Res. Organ. Behav. 18, 1–74.

[ref74] Weiss H. M. Suckow K. Cropanzano R. (1999). Effects of justice conditions on discrete emotions. J. Appl. Psychol. 84, 786–794. doi: 10.1037/0021-9010.84.5.786

[ref75] Yuan D. Hu M. Yao N. Zhong H. Xiao Y. Zhou X.. (2024). Effects of perceived stress on turnover intention of female healthcare staff: a serial multiple mediation model. BMC Public Health 24:1198. doi: 10.1186/s12889-024-18654-z, PMID: 38685094 PMC11059584

[ref76] Yukl G. (2008). How leaders influence organizational effectiveness. Leadership Q. 19, 708–722. doi: 10.1016/j.leaqua.2008.09.008

[ref77] Zhang X. X. Hao X. L. Wang B. C. (2024). Multiple realization paths of the trickle-down effect of leader resilience. Nankai Bus. Rev. 27, 28–39+50. (In Chinese)

[ref78] Zhang K. L. Tang N. Y. (2016). Being honest in organizations: the antecedents of outcomes of employees’ error admission. Nankai Bus. Rev. 19, 36–48. (In Chinese). doi: 10.3969/j.issn.1008-3448.2016.06.005

[ref79] Zhang K. L. Yin Q. Tang N. Y. (2021). Under what circumstances do employees report their errors? -a perspective of secret sharing. Adv. Psychol. Sci. 29, 1149–1162. (In Chinese). doi: 10.3724/SP.J.1042.2021.01149

[ref80] Zhou X. Cheng T. (2020). Can leader’s tolerance behavior foster employee’s proactivity? A cross-level analysis. Econ. Manag. 42, 109–124. (In Chinese). doi: 10.19616/j.cnki.bmj.2020.01.007

